# Hyaluronic Acid-Based Theranostic Nanomedicines for Targeted Cancer Therapy

**DOI:** 10.3390/cancers12040940

**Published:** 2020-04-10

**Authors:** So Yun Lee, Moon Sung Kang, Woo Yeup Jeong, Dong-Wook Han, Ki Su Kim

**Affiliations:** 1 Department of Organic Materials Science and Engineering, College of Engineering, Pusan National University, 2 Busandaehak-ro 63 beon-gil, Geumjeong-gu, Busan 46241, Korea; nanobiosyl@gmail.com (S.Y.L.); duq5315@naver.com (W.Y.J.); 2 Department of Cogno-Mechatronics Engineering, College of Nanoscience and Nanotechnology, Pusan National University, 2 Busandaehak-ro 63 beon-gil, Geumjeong-gu, Busan 46241, Korea; mskang7909@gmail.com

**Keywords:** hyaluronic acid, cancer therapy, anticancer agent, theranosis, nanomedicine, receptors

## Abstract

Hyaluronic acid (HA) is a natural mucopolysaccharide and has many useful advantages, including biocompatibility, non-immunogenicity, chemical versatility, non-toxicity, biodegradability, and high hydrophilicity. Numerous tumor cells overexpress several receptors that have a high binding affinity for HA, while these receptors are poorly expressed in normal body cells. HA-based drug delivery carriers can offer improved solubility and stability of anticancer drugs in biological environments and allow for the targeting of cancer treatments. Based on these benefits, HA has been widely investigated as a promising material for developing the advanced clinical cancer therapies in various formulations, including nanoparticles, micelles, liposomes, and hydrogels, combined with other materials. We describe various approaches and findings showing the feasibility of improvement in theragnosis probes through the application of HA.

## 1. Introduction

Cancer is a leading cause of death in the United States and numerous other parts of the globe. The new cancer cases worldwide are predicted to increase from approximately 14 million in 2012 to more than 22 million in 2030. The number of deaths from cancer is expected to continue rising and to reach 13.1 million in 2030 [1,2,3,4]. Therefore, successful cancer treatment is one of the most important goals of current medical science. Recently, advances in nanotechnology have made it possible to understand the fundamental biology of cancer, and to develop new and promising therapies [5]. Various materials and methods have been reported for producing drug carriers that can protect and deliver therapeutic molecules to tumors [6].

**Figure 1:**
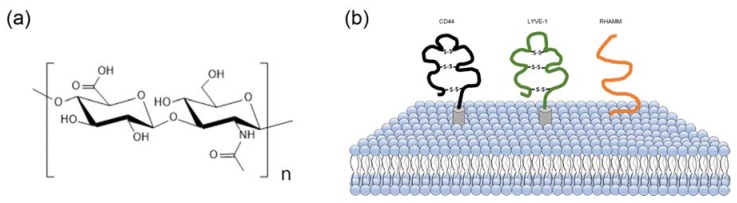
(**a**) The structure of hyaluronic acid, composed of alternating units of D-glucuronic acid and N-acetyl-D-glucosamine, (**b**) hyaluronic acid receptors in the cell: cluster of differentiation 44 (CD44), lymphatic vessel endocytic receptor (LYVE-1), and the receptor for hyaluronic acid-mediated motility (RHAMM)**.**

Hyaluronic acid (HA) is a natural mucopolysaccharide comprising alternately repeating disaccharide units of D-glucuronic acid and N-acetyl-D-glucosamine (Figure 1a) and is the main constituent of the extracellular matrix (ECM). The HA plays a significant role in cell growth and in maintaining the structural stability of tissue [7,8,9]. In the body, these structural roles are affected by their hydrodynamic properties and interactions with other ECM components. As HA is biocompatible, non-immunogenic, non-toxic, biodegradable, chemically modifiable, highly hydrophilic, and can absorb water, producing viscoelastic gel, HA has attracted considerable interest from researchers for biomedical applications, including drug delivery systems [10,11].

In addition, there are several overexpressed HA binding receptors in cancer cells compared to normal cells, such as cluster of differentiation 44 (CD44) [12,13,14], lymphatic vessel endocytic receptor (LYVE-1) [15], and the receptor for hyaluronic acid-mediated motility (RHAMM) [16] (Figure 1b). These receptors can offer selective tumor targeting. The role of CD44 in the interactions between HA and specific cells has been extensively explored. The CD44 family of proteins belongs to transmembrane glycoproteins and play a crucial role in extracellular adhesion, cell activities, and signal transduction [17]. The CD44 receptor is involved in tumor invasion and metastasis in cancer cells, and has been associated with the cellular adhesion process, including aggregation and migration in normal biological systems [18,19].

There are some differences between normal cells and tumor cells concerning the HA and CD44 receptors. In normal tissue, the CD44 receptor is endogenously expressed with low levels on different cells and requires activation [20]. However, tumor-derived cells do not require an activation process because the expressed CD44 receptor has a high affinity. In this case, HA binding and internalizing are possible without additional procedures. These interactions can encourage tumor cell migration, which is linked with HA levels; HA levels are high at the edges of rapidly growing tumors [21].

As mentioned above, CD44 receptors are overexpressed on the surfaces of various tumor cells, including breast cancer [22,23] and lung cancer [12,24], therefore, CD44 can be utilized as a cancer-targeting biomarker. RHAMM is another well-known HA-specific receptor that mediates cell proliferation and migration and is poorly expressed in the majority of common normal tissues. Conversely, RHAMM shows increased expression in tumor cells, which is related to metastases [25,26].

HA-based drug delivery carriers provide several advantages. First, HA can improve the stability of anticancer drugs in physiological conditions [27]. Secondly, HA can solve the drawbacks of current anticancer drugs, such as low specificity, via several overexpressed receptors that selectively bind to HA [28,29]. Finally, HA can be chemically modified through functional groups. The carboxyl groups on the glucuronic acid unit and the primarily hydroxyl groups on the N-acetyl-D-glucosamine unit are commonly used groups for chemical modification to obtain HA derivatives [30,31].

In this review, we discuss the approaches that utilized various formulations of HA to design drug carriers and advances in HA-based drug delivery systems for improved cancer treatment. Moreover, we present a brief overview of the recent findings and progression in the research to show the promising aspects of HA-based cancer therapies.

## 2. HA–Drug Conjugates

In anticancer therapy, there are various treatment methods, the most basic of which is chemotherapy using anticancer drugs, such as paclitaxel (PTX), doxorubicin (DOX), cisplatin (cis-diamminedichloroplatinum (II) or CDDP), SN-38, etc. For cancer therapy, these drugs administered into the body can act on cancer cells or tissues by reducing cell viability or expediting a specific immune reaction for the elimination cancer tissues. However, these effects can cause damage to normal cells as well as cancer cells, resulting in various side effects. Furthermore, they can be easily eliminated by physiological or immune reactions in the body, since these drugs are external substances administered into the body.

**Figure 2:**
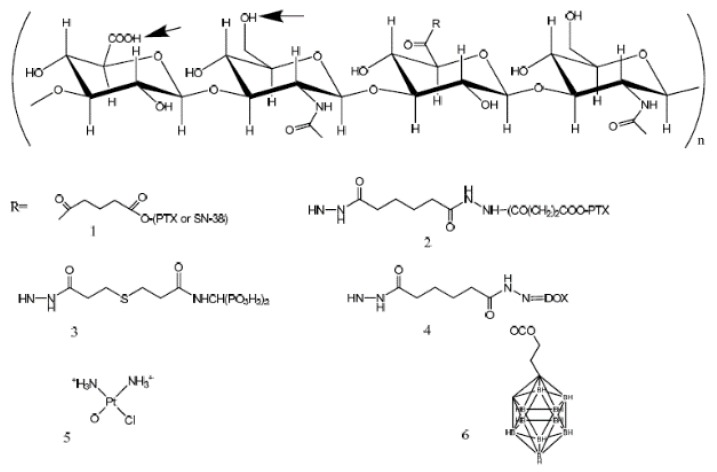
Hyaluronic acid (HA) and small molecule conjugates. Arrows indicate chemical modification sites of HA and R is link between HA and the anticancer drugs. Anticancer drugs are conjugated with HA at the carboxyl and hydroxyl groups of HA through ester linkages or amide linkages [32].

Therefore, it is necessary that administered drugs act on the cancerous tissue without affecting other normal cells and maintain a stable state in in vivo microenvironments until they are delivered. To this end, HA, with the various advantages discussed earlier, has been applied for drug delivery of anticancer drugs [32,33,34,35,36]. In general, anticancer drugs have been conjugated with HA at the carboxyl group and hydroxyl group through functional groups of the drug itself or conjugate linkers, such as ester linkers and amide linkers (Figure 2).

### 2.1. HA-Conjugated Paclitaxel

Paclitaxel (PTX) is the most well-known anticancer drug for the treatment of numerous tumors, such as breast, ovarian, and melanoma cancer. However, it has several limitations due to poor water solubility, nonselective toxicity, and inactivity against drug-resistant cell lines [37]. For these reasons, HA conjugation can offer improvements to these problems.

Rosato’s group synthesized ONCOFID^TM^-P, a novel PTX-HA (MW 200 kDa) conjugate, using 4-bromobuyric acid, which can form two ester linkages between PTX and HA, for treating superficial bladder cancer [37,38]. A subsequent imaging biodistribution analysis of ^99^mTc-radiolabeled ONCOFID^TM^-P by the intravascular method was conducted. As a result, abdominal and gastroenteric tissue after injection showed that the radiolabeled conjugate remained in the cancer tissue. Therefore, these methods can be relevant to local treatment for bladder cancer cells.

Xin et al. conjugated PTX with HA using amino acids between PTX and HA (MW 9.8 kDa), which showed more stability in in vivo conditions [39]. Amino acid linkers were conjugated to the carboxyl group with the hydroxyl group of PTX. Then, intermediates were conjugated to the amino group of the amino acid with the carboxyl group of HA, using carbodiimide activation, and the HA-PTX prodrug was successfully synthesized. Prodrugs exhibited increased cytotoxicity compared to free paclitaxel against the MCF-7 cell line in an in vitro model. Mittapalli et al. conjugated HA (MW 4~5 kDa) and PTX using a polyethylene glycol (PEG)-linker-forming ester linkage, and applied this conjugation to human breast cancer cells (MCF-7) [40]. As a result, the HA–PTX treated group showed that nanoconjugate remained in the body for a longer time compared with the pure PTX treated group. Therefore, HA–PTX improved the drug efficacy in a preclinical model of breast cancer. Moreover, Zhong et al. developed paclitaxel prodrug micelles based on HA (MW 9.5 kDa)-b-dendritic oligoglycerol nanoparticles (HA-dOG-PTX-PM NPs) encapsulating fluorescence dye (DiR) for effective targeting and treatment of xenografted human breast cancer in vitro and in vivo. Through in vivo biodistribution analysis for 48 h, it was confirmed that DiR was significantly accumulated in tumor tissue compared to other organs at 4 h post injection. This demonstrated that a theranostic system via these NPs is possible based on tumor-targetability through HA conjugation and the anti-cancer effect of PTX and bio-imaging through DiR loading [41].

### 2.2. HA-Conjugated Doxorubicin

Doxorubicin (DOX) is one of the most well-known anthracycline antibiotics utilized for cancer therapy. Despite its wide clinical use for chemotherapy, it can cause a dose-dependent toxicity. Oommen et al. prepared HA-DOX conjugates via covalent linkages between DOX and HA. As HA plays a critical role in cancer targeting, such polymer–drug conjugates can be an effective method to treat metastatic tumors. The HA-DOX particles are stable in serum, as it is known that hyaluronidase activity is significantly reduced in the blood serum of cancer patients. This method could also reduce the in vivo toxicity caused by early release of the drug in vivo [42].

Cai et al. developed HA-DOX conjugates using the HA (MW 35 kDa)-adipic acid dihydrazide (ADH) derivative [43]. In this study, the HA-DOX treatment group showed delayed cancer progression for approximately 10 weeks, and compared with the free DOX treatment group, increased the animals’ survival. HA-DOX showed significant efficacy combined with decreased toxicity, achieving a complete pathologic tumor response. In addition, an antitumor theranostic system using HA (MW 100 kDa)-DOX conjugates was reported by Kim et al. [44]. The HA-DOX conjugates were prepared by chemical reaction between carboxylic groups of HA and amine groups of DOX and formed micelle-like NPs through self-assembling. In this research, the antitumor effect of HA-DOX micelles in in vitro anti-proliferation tests of cancer cells and successful formation of the micelles coated with gold (Au) half-shells to take advantage of NIR-absorbing and electromagnetic properties of Au half-shell for theranostic applications to cancer treatment were demonstrated.

### 2.3. HA-Conjugated Cisplatin

CDDP is the drug that can be applied to most cancer therapy. However, there are serious side effects such as neurotoxicity, myelosuppression, and nephrotoxicity, which limits its use. To improve tumor targeting and avoid side effects, several approaches have been developed for conjugation with HA. In 2008, Cai et al. reported HA-CDDP conjugates produced by hydrolysis of chloride on CDDP and replacement with carboxylates on HA (MW 35 kDa) using silver nitrate (AgNO_3_). The resulting conjugates exhibited an increase in local concentration in the drain lymph node basin without decisively affecting the target organ in an intralymphatic delivery model [45]. Xie et al. also demonstrated that the lung instillation of HA (MW 35 kDa)-CDDP conjugates increased Pt accumulations in the lung tissue and peripheral lymph nodes compared to conventional CDDP i.v. infusion and show a sustained-release plasma profile [46]. Moreover, compared with pure CDDP, HA conjugated CDDP has a targeting effect on cancer cells, which can selectively increase the concentrations in cancer cells and tissues. Cohen et al. applied HA-CDDP to head and neck squamous cell carcinoma (HNSCC), and confirmed a significant improvement in the antitumor efficacy, with a lower toxicity compared to free CDDP [47]. In the study, each experimental group was measured for weight loss and given a body conditioning score. The HA-CDDP treated group exhibited the least weight loss compared to the CDDP-only treated group. In addition, the body score was decreased below 2 points in all control groups, but not in any HA-CDDP treated group. Therefore, the HA conjugated groups had no toxic problems compared to the control group.

## 3. HA-Based Nanomaterials

HA has several functional groups that enable the encapsulation of active pharmaceutical ingredients or adsorption of macromolecular substances [48]. On the other hand, nanomaterials possess excellent potential for cancer therapy owing to their unique physicochemical properties [49]. Therefore, several approaches have been developed to synthesize functional nanomaterials using HA [50,51,52]. HA itself can be synthesized into conjugated polymers, such as dendrimers, micelles, liposomes, and hydrogels. Furthermore, HA can be incorporated into nanomaterials for enhanced water solubility, biocompatibility, and targetability by their specific binding to CD44 overexpressed cancer cells. Moreover, HA does not induce immune reactions and is biocompatible, suggesting that HA can be potentially applied for improved theragnosis probes for cancer treatment.

### 3.1. Dendrimers

Dendrimers are highly branched synthetic polymers with layered structures around an internal core. Each branch that extends from the internal core has different terminal groups, which determine the characteristics of the dendrimer [53]. Dendrimers have uniform and controllable size/chemical composition and a high loading capacity of payloads but have limited chemical synthesis methods [54]. Therefore, many studies focused on developing new synthesis methods to induce various modifications of the terminal groups of HA dendrimers for cancer therapy.

**Figure 3:**
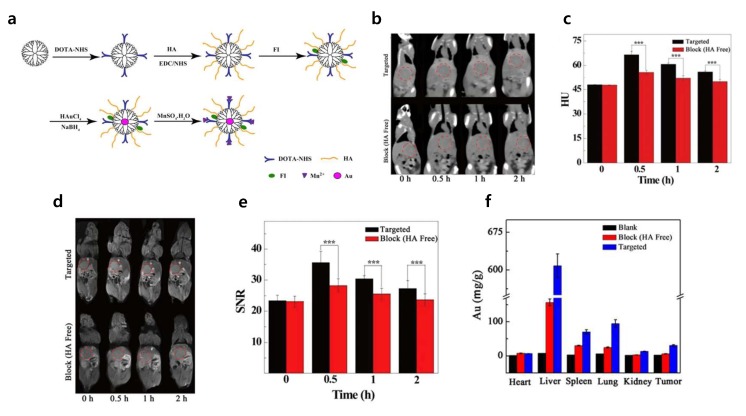
(**a**) Schematic diagram of the synthesis of {(Au^0^)_100_G5.NH_2_-FI-DOTA(Mn)-HA} nanoparticles (NPs). (**b**) In vivo CT images and (**c**) CT values (Hounsfield units, HU) of orthotopic liver tumors at different times after a 0.3 mL intravenous injection of a {(Au^0^)_100_G5.NH_2_-FI-DOTA(Mn)-HA} NP solution (0.3 mL in PBS, [Au] = 120 mM). (**d**) In vivo MR images and (**e**) signal-to-noise ratio (SNR) of orthotopic liver tumors at different times after an intravenous injection of 0.3 mL of a {(Au^0^)_100_G5.NH_2_-FI-DOTA(Mn)-HA} NP (300 μg Mn) solution in PBS. (**f**) Biodistribution in the major organs of the mice and tumors at 24 h after the intravenous injection of a PBS solution containing {(Au^0^)_100_G5.NH_2_-FI-DOTA(Mn)-HA} NPs (0.3 mL in PBS, [Au] = 120 mM). Reproduced from reference [55], copyright open access by Creative Commons Attribution 4.0 International License 2016.

Wang et al. encapsulated Au NPs with a G5.NH_2_ dendrimer, functionalized with an Mn chelator, 1,4,7,10-tetraazacyclododecane-1,4,7,10-tetraacetic acid (DOTA), fluorescein isothiocyanate (FI), and HA with Mw 6 kDA {(Au^0^)_100_G5.NH_2_-FI-DOTA(Mn)-HA}. The prepared {(Au^0^)_100_G5.NH_2_-FI-DOTA(Mn)-HA} NPs endowed encapsulated Au NPs with several advantages, such as enhanced water solubility, stability under different conditions, and cytocompatibility. The prepared NPs were utilized as an imaging probe for tumor cell imaging [55]. The HA modified Au dendrimers were accumulated 2-fold higher in tumors compared to pure Au-dendrimers, indicating the tumor specificity of HA (Figure 3) [55].

On the other hand, there have been many studies utilizing dendrimer-based drug-delivery strategies exploiting HA as a targeting ligand for the targeted delivery of drugs for cancer. Kesharwani et al. successfully reduced the cationic surface charge of native poly(amidoamine) (PAMAM) by conjugation of HA (10 kDa) with the peripheral amino groups of PAMAM dendrimers through N-(3-dimethylaminopropyl)-N′-ethylcarbodiimide (EDC) coupling chemistry [56]. Then, 3,4-difluorobenzylidene curcumin (CDF) was encapsulated in HA-PAMAM dendrimers and efficiently transferred into the nucleus of tumor cells by CD44 receptor mediated endocytosis [56].

### 3.2. Micelles

HA also can be formed into polymeric micelles. Polymeric micelles are synthesized by the self-assembly of amphiphilic copolymers in aqueous solution and have a spherical structure with hydrophilic heads at the shell and hydrophobic tails at the core [57]. Owing to their amphiphilic structure, micelles can effectively load water-insoluble drugs and slow down in vivo degradation processes such as uptake by the reticuloendothelial system and blood clearance [57]. Moreover, drug release can be controlled by external stimuli such as pH, temperature, enzymes, ultrasound, etc. [58,59,60,61]. The advantages of micelles for drug delivery are the high dissolution capacity, stability, sustained release, long-term circulation, and the ability to remain in the tumor for a long time due to the enhanced permeability and retention effect (EPR effect), which is the abnormal molecular and fluidic dynamic of certain macromolecules and lipids caused by the specific nature of tumor tissues, such as imperfect vascularization, lack of lymphatic drainage, wider lumen, and so on.

Lee et al. synthesized hydrophobic poly(lactic-co-glycolic acid) (PLGA) multi-cores for doxorubicin encapsulation by the self-assembly of PLGA grafted HA (low and high MW: 17 and 64 kDa, respectively) copolymers. DOX-loaded HA-PLGA micelles exhibited enhanced cellular uptake and greater cytotoxicity to human colon cancer cells (HCT116) [62]. Redox-sensitive amphiphilic HA-deoxycholic acid conjugates were developed for the targeted intracellular delivery of paclitaxel [63]. The conjugates were self-assembled into nanosized micelles in aqueous media and exhibited high drug loading capacity and encapsulation efficiency [63]. HA-deoxycholic acid micelles were sufficiently stable in physiologic conditions but quickly disassembled in the presence of a reducing agent, suggesting that their drug release can be controlled by redox-sensitivity [63].

Similarly, pH-responsive HA-g-poly(L-histidine) (HA-PHis) copolymer micelles were developed with HA (MW 11 kDa) for intracellular DOX delivery [64]. DOX was efficiently loaded into self-assembled HA-PHis-conjugated micelles in aqueous conditions [64]. These micelles were up-taken to the cancer cells by receptor-mediated endocytosis, and drug release could be controlled by pH differences of the intra/extra cellular environments [64].

### 3.3. Liposomes

Liposomes are vesicles composed of 50–100 nm phospholipid bilayers, similar to biological membranes. The unique amphiphilic properties of liposomes make it possible to transport hydrophilic drugs in aqueous solutions and to dissolve hydrophobic drugs through membranes [65]. In addition, liposomes are synthetic carriers approved by FDA (Food and Drug Administration) and have excellent circulatory, penetration, and controllable diffusion properties depending on their chemical composition [66,67]. However, polymeric liposomes generally have poor in vivo stability and it is difficult for them to maintain their loaded drugs until they reach the targeted site [67].

Taetz et al. synthesized cationic HA modified 1,2-dioleoyl-3-trimethyllammoniumpropane/ dioleoylphosphatidylethanolamine (DOTAP/DOPE) liposomes using an ethanol injection method for the targeted delivery of anti-telomerase small interfering RNAs (siRNAs) to CD44+ lung cancer cells. The presence of HA upregulated siRNA delivery into lung tumor tissues by the enhancement of siRNA conjugation, the protection of siRNA in the presence of RNase V1, and complex stability in the presence of serum [68]. To improve the cancer cell targetability, dual-functionalization, such as pH-responsive cell penetration peptide (CPP)-HA core-shell liposomes, can be utilized [69]. The HA shell primarily delivers liposome complexes to the tumor site and is then removed by hyaluronidase to expose the inner pH-responsive CPP to promote cellular uptake.

## 4. HA-Coated NPs

Inorganic nanomaterials such as Au nanoparticles, quantum dots, magnetic nanoparticles, ceramic nanoparticles, and carbon-based NPs have attracted considerable attention in the treatment of cancer [70]; however, these systems have drawbacks, including cytotoxicity and non-cell specificity. Therefore, surface modification with biopolymers has been widely investigated to develop further functionalized cancer theranosis probes [71,72,73]. Likewise, surface coating with HA on inorganic NPs has been studied for biocompatibility and biofunctionality, as well as targetability.

### 4.1. HA-Coated Au NPs

Au NPs can be potentially used in selective photothermal therapies induced by lasers, owing to their surface plasmon resonance. Au NPs are biocompatible and can easily provide surface modification due to their ability to bind amine and thiol groups, optical properties that can be adjusted according to size and shape, and optical quenching ability [74,75,76]. However, since Au NPs are typically cleared slowly, they exhibit longer-term whole-body retention in some cases [77]. Other challenges of the application of Au NPs are their low stability, reactivity, and capacity to load hydrophilic drugs. Therefore, the surface functionalization of Au NPs to solve these problems is currently one of the most intensively researched topics.

HA coatings have several advantages, such as antifouling effects on the prevention of protein adsorption and opsonization due to the hydrophilic and polyanionic characteristics in physiological environments [78,79,80]. HA-conjugated Pheophorbide-A (PheoA) and Au NPs could serve as multifunctional theranostic nanoagents for photodynamic and photothermal therapy. Thiolated HA (MW 7 kDa) was conjugated with PheoA first, then Au NPs were encapsulated within PheoA-HA conjugated by the Au–thiol reaction. This PheoA-HA-Au nanoagent showed excellent colloidal stability under physiological conditions and restored photoactivity in intracellular environments.

Furthermore, tumor specificity and therapeutic efficacy in tumor-bearing mice were significantly increased [81]. On the other hand, HA-Au NPs can be used as protein drug delivery carriers. Lee et al. investigated HA-Au NPs by chemical binding of thiolated HA (MW 12 kDa) and physical binding of interferon α to Au NPs (HA- Au NP/IFNα). Prepared HA-Au NP/IFNα showed enhanced stability and efficiency compared to Au/IFNα and PEGylated IFNα, which is a conventional IFNα carrier [82].

### 4.2. HA-Coated Quantum Dot

A quantum dot (QD) is a colloidal nano-sized single crystal exhibiting fluorescence. The center of a QD is generally composed of semiconductor materials, such as CdSe, CdTe, CdS, PbSe, ZnS, ZnSe, GaAs, GaN, InP, and InAs. QDs have excellent light stability and show a tunable emission spectrum and high quantum yield depending on their size and composition. In addition, QDs have been widely used in biological applications as imaging contrast agents and labeling agents due to their low photobleaching and low photo- and chemical degradation [83]. However, clinical applications of QDs are still limited because of their composition of toxic heavy materials and low water solubility [83,84,85].

**Figure 4:**
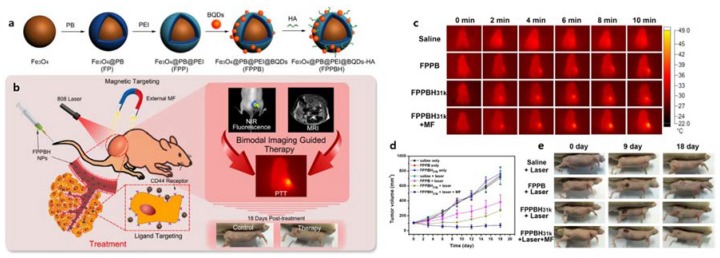
Schematic illustration of the (**a**) Fe_3_O_4_@PB@PEI@BQDs-HA (FPPBH) NPs fabrication procedure and (**b**) NIR fluorescence/MR bimodal imaging-guided cancer photothermal therapy (PTT) through intravenous injection. (**c**) Infrared thermal images of HeLa-bearing nude mice under the 808 nm laser irradiation taken at different time intervals. (**d**) Therapeutic effectiveness expressed as tumor volume in each group after treatment in HeLa-bearing nude mice. Data shown as mean SD, n = 8. (**e**) Photographs of representative mice of the four different groups taken before and after treatment for 9 and 18 days. Reproduced from reference [87], copyright open access by Ivyspring 2017.

Wang et al. investigated HA-coated QD as a CD44+ cancer cell-targeted imaging probe. A cysteamine-modified HA polymer was employed to coat CdSe (CdZnS) QDs through a convenient one-step reverse micelle method. Prepared HA-QDs showed enhanced stability in PBS and fluorescence stability, and excellent targeting ability to CD44+ breast cancer cells without cytotoxicity, indicating that the stability and low-toxicity of QDs could be achieved through an HA coating [86]. Meanwhile, Yongbo et al. prepared QDs coated by HA-magnetic Prussian Blue conjugates (HA-PB@QDs) for cancer theranosis (Figure 4). The targeting efficiency of HA-PB@QD to lung cancer cells was enhanced by the coexistence of a magnetic core and CD44 ligand HA, which was found to significantly improve the specific uptake by CD44-overexpressed HeLa cells upon external magnetic fields. Moreover, enhanced in vivo photothermal therapy (PTT) efficacy upon NIR laser illumination was observed, indicating the tumor growth inhibition was more than 89.95% [87].

### 4.3. HA-Coated Superparamagnetic Iron Oxide NPs

Superparamagnetic iron oxide NPs are characterized by biocompatibility, controllable size and shape, and clinically available contrast agents for MRI. However, their clinical application is quite limited due to the poor stability under aqueous conditions, such as the tendency to aggregate due to the large surface area and strong magnetic properties [88]. In addition, magnetic NPs with superparamagnetic properties should be able to maintain a colloidal state for a long time and change surface properties through covalent bonds and be well dispersed in water. However, finding the ideal combination is challenging.

To solve these problems, HA coatings have emerged as a novel method. To prepare HA-coated iron oxide NPs, polyethyleneimine (PEI) drug conjugates were investigated [89]. PEI stabilized Fe_3_O_4_ NPs were prepared via a one-pot hydrothermal method, and modified with HA and fluorescein isothiocyanate (HA/FI-Fe_3_O_4_ NPs) to be utilized as a cancer diagnosis probe. The HA/FI-Fe_3_O_4_ NPs were water-dispersible and cyto/hemo-compatible. The prepared NPs showed enhanced cellular uptake of HeLa cells through CD44 receptor-mediated active targeting pathways and exhibited intracellular green fluorescence.

### 4.4. HA-Coated Carbon-Based NPs

A wide variety of different nanomaterials based on the allotropic forms of carbon, such as nanotubes, nanohorns, and nanodiamonds, have been explored towards different biomedical applications [90]. To enhance the cancer cell specificity and water stability of carbon-based nanoparticles, HA coatings were performed with several methods. A reduced graphene oxide (rGO) with an HA-based amphiphilic polymer was produced by grafting HA onto poly(maleic anhydride-*alt*-1-octadecene) (PMAO) [91]. On the other hand, HA-coated rGO nanosheets were obtained by coating with cholesteryl hyaluronic acid (CHA), which was synthesized using cholesteryl-2-aminoethylcarbamate (CAEC) with the carboxyl group of HA [92]. Both nanoprobes exhibited improved stability and cytocompatibility and could potentially be utilized for targeted cancer PTT. Other carbon-based nanomaterials, such as single-walled carbon nanotubes (SWNT), graphene oxide (GO), and fullerene (C_60_) for anticancer activity, could be HA-surface modified. Prepared HA-SWNT, HA-GO, and HA-C_60_ showed significantly enhanced water solubility, biocompatibility, and tumor-targeting capacities [93].

## 5. HA-Based Hydrogel

Hydrogels are three-dimensional hydrated polymeric networks, formed from crosslinked polymer chains, capable of absorbing water by 10–20% up to thousands of times their dry weight [94]. Their highly porous structures enables drug release in a controlled manner depending on the bulk loading of drugs into the gel matrix and the diffusion efficiency of molecules inside the gel network. However, HA hydrogels have some drawbacks as drug delivery probes due to their susceptibility to degradation and low mechanical properties [48,95]. Therefore, chemical modifications, covalent crosslinking, and gelling agents are always needed in order to use HA-based hydrogels as drug delivery systems [96]. In this section, several types of HA hydrogels are introduced for the controlled release of loaded drugs.

### 5.1. In situ Crosslinked HA Hydrogels

The treatment of localized infections within the body without surgery can be achieved with injectable drug delivery systems. In situ crosslinked hydrogels can be easily injected but maintain a viscose matrix after injection to enable a local drug concentration for a desired time. Recently, an enzymatic crosslinking was used with in situ crosslinking of hydrogels. An HA-tyramine conjugate (HA-Tyr) (MW 90 kDa) was investigated as an injectable and biodegradable drug carrier [97]. HA-Tyr was developed through the oxidative coupling reaction of the Tyr moieties, catalyzed by hydrogen peroxide (H_2_O_2_) and horseradish peroxide (HRP). The advantage of enzymatic crosslinking using H_2_O_2_ and HRP is the concentration-dependent tuning of the hydrogel stiffness and gelation rate [97].

Rapid gelation was achieved by an optimal concentration of HRP that could effectively encapsulate the proteins within the hydrogel network and thus prevent the undesired leakage of proteins into the surrounding tissues after injection [98]. HA-Tyr also can be used for liver cancer therapy [99]. Interferon-α2a (IFN-α2a) was incorporated into prepared HA-Tyr, which revealed a greatly inhibited proliferation of liver cancer cells and induced apoptosis. Moreover, IFN-α2a incorporated in HA-Tyr highly increased the angiogenesis of mouse tumor tissues, suggesting that HA-Tyr modification can be developed as a method to deliver IFN-α2a into liver tumor tissues [99].

However, there are several studies that indicate that the potential risk of HA in situ hydrogels should be studied. A platinum nanoparticle/HA gel (PtNP/gel) was loaded into a chemically crosslinked HA hydrogel and applied for the local therapy of ovarian cancer. The gel was composed of adipic dihydrazide modified HA (HA-ADH) and oxidized HA (HA-CHO), which can be applied as a liquid and instantly crosslinked via the hydrazone links to form a gel as they mix. The PtNP/gel was maintained in the peritoneal cavity over 4 weeks and the PtNPs release was retained for a prolonged time and locally delivered to CD44+ ovarian cancer cells by receptor-mediated endocytosis. However, the PtNP/gel did not show enhanced anti-cancer efficacy, but rather slightly increased tumor bundles, which was suspected to be due to the effects of the potential involvement of residual empty carriers and degradation products. This study warns that HA carriers may have unwanted biological effects on the residual targets after the drug is released [100].

HA-epigallocatechin-3-O-gallate conjugated injectable hydrogels (HA-EGCG) were synthesized by thiol-mediated reactions, and they exhibited in vivo prolonged properties. HA-EGCG conjugates with tunable degrees of substitution were synthesized by the nucleophilic addition reaction between EGCG quinone and thiolated HA under mild conditions. When injected subcutaneously in mice, HA-EGCG hydrogels were retained much longer than HA-tyramine hydrogels owing to the hyaluronidase inhibitory activities of EGCG [101].

### 5.2. Thermosensitive HA Hydrogel

Many polymers exhibit a temperature-responsive phase transition property. The common characteristic of thermosensitive polymers is the presence of hydrophobic groups, such as methyl, ethyl, and propyl groups. Thermosensitive polymers, such as poly(N,N-isopropylacrylaminde) (PNIPAAm), feature lower critical solution temperature (LCST) in the body temperature range and are widely clinically used [102]. Thermosensitive HA hydrogels for the delivery of drugs to locally treat tumors were investigated.

HA conjugated with dopamine (HA-DN) (MW 130 kDa) was mixed with thiol end-capped pluronic F127 copolymer (Plu-SH) to produce lightly cross-linked HA/pluronic composite gel structures based on Michael-type catechol-thiol addition reactions. The prepared HA/pluronic hydrogels exhibited temperature-dependent rapid and reversible sol–gel phase transition behaviors, enabling the hardening of the hydrogel at body temperature [103]. Thermosensitive injectable hydrogels composed of a nanocomplex of Dox and HA for the local treatment of cancer disease were also developed. An HA-Dox nanocomplex was synthesized by the addition of divalent metal ions of Mg and was mixed with pluronic F127 to form thermosensitive hydrogels. The prepared hydrogel efficiently inhibited C26 colon cancer cell growth and selectively targeted the lymphatic system by the specific affinity of HA to the lymphatic system [104].

### 5.3. pH-Sensitive Hydrogels 

The pH-sensitive hydrogels possess pendant acidic groups (e.g., carboxylic and sulfonic acid) or basic groups (e.g., ammonium salts) enabling proton absorption or release in accordance with the external pH change [105]. The pH-sensitive hydrogels are widely used as oral medication. The pH values in the mouth, stomach, and intestine are significantly different, therefore, drug release can be efficiently controlled. Moreover, pH values of tumor tissues are generally lower than normal tissues because the glycolysis of tumor cells can cause acidification [106]. Thus, several synthesis approaches for pH-sensitive hydrogels based on HA have been attempted for drug delivery and, furthermore, cancer treatment. Nucleobase pairing was investigated to synthesize pH-sensitive HA hydrogel cross-linking via hydrogen bonding, which was achieved under physiological conditions by cytosine and guanosine complementary base pairing, with 1,6-hexamethylenediamine (HMDA) as a bridging unit between nucleobase and HA (HA-HMDA-C, -G, -C/G according to paired nucleobases [107]). The prepared hydrogel possessed a suitable gelling time, good rheology properties, high swelling ratio, biodegradability, effective drug loading capacity, and sustained drug release ability under physiological conditions [107].

In 2015, Khatun et al. developed a drug delivery system using light-responsive graphene, DOX, and pH-sensitive disulfide-linked HA (MW 7000 kDa) to form a nanoscale hydrogel called nanogel for theranostics of cancer. The prepared nanogel exhibited pH-dependent drug release and enhanced tumor accumulation through a receptor-mediated pathway in optical imaging in vitro and in vivo. Furthermore, nanogels were effective in killing a human lung cancer cell line (A549) and simultaneously limited the toxicity in normal cells [108].

**Figure 5:**
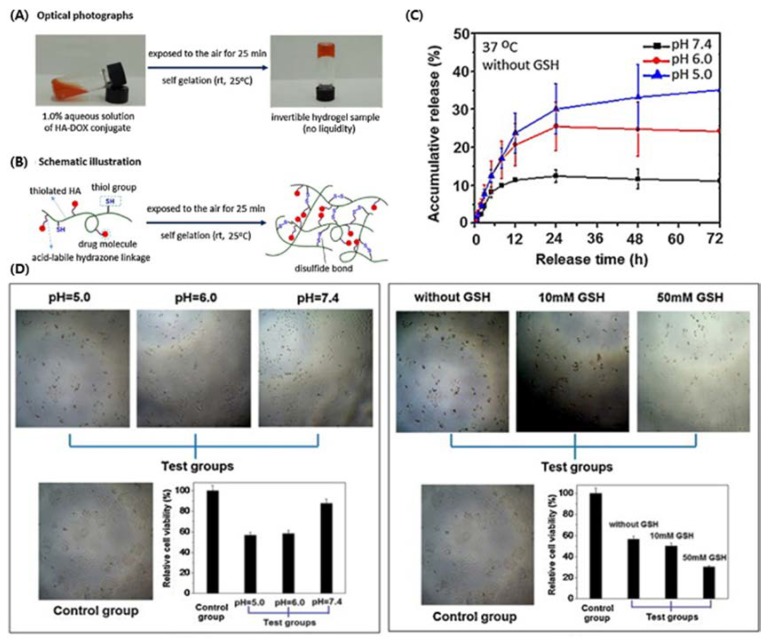
Dual stimuli-responsive drug release hydrogel. Optical photographs (**A**) and schematic illustration for the self-gelation (**B**) of aqueous 1.0 wt% HA–DOX conjugate solution when exposed to the air at room temperature (23 °C), and (**C**) pH-triggered drug release profiles of HA–DOX conjugate hydrogel. The photographs (×5) and relative viabilities of human nasopharyngeal carcinoma CNE2 cells: (**D**) treated with the release media of HA–DOX conjugate hydrogel under various pH conditions without glutathione (GSH) and treated with the release media of HA–DOX conjugate hydrogel in the absence and presence of GSH (10 and 50 mmol/L) when the pH value was kept at 5.0. Individual PBS (pH 7.4) was used as the control. Reproduced from [109], copyright permission by Elsevier 2017.

In addition, dual stimuli-responsive hydrogels loaded with DOX were prepared with thiolated HA (MW 320 kDa) crosslinked by an acid-labile hydrazine linker. The hydrogel exhibited a sustained release of DOX in both pH- and reduction-response modes under conditions that mimic the intracellular environments of cancer cells, and effectively inhibited human nasopharyngeal carcinoma CNE2 cells (Figure 5, [109]).

## 6. Conclusions and Perspectives

Hyaluronic acid is one of the most attractive molecules in the field of developing anticancer drug delivery carriers and imaging agents due to its physicochemical properties that provide various benefits in drug delivery systems. This review summarized the recent progress made with the HA-drug conjugates, HA-based nanomaterials (dendrimers, micelles, and liposomes), hydrogels, and HA-coated NPs, which are used as drug delivery carriers for cancer treatment. Various chemical approaches have been studied to develop HA-based anti-cancer drug delivery methods.

In general, the HA conjugates improved the solubility of hydrophobic drugs and the targeting efficiency to tumor cells with minimum toxicity. From the results described herein, conjugated polymers prepared by themselves or incorporated into other nanomaterials enhanced the solubility, stability, biofunctionality, and ability to target CD44, which is overexpressed in tumor cells. Many studies demonstrated that applying HA can improve theranosis probes for cancer therapy. Moreover, surface coating with HA can be applied to address the issues of inorganic NPs in cancer therapy, as well as provide a more efficient theranostic system.

However, considering such factors as drug loading, in vivo properties, and clinical safety, few studies have been directly compared to clinically available products that provide the best balance. In addition, before attempting clinical applications, well-proven studies such as biodistribution, toxicity, and availability in physiological conditions are essential to estimate the feasibility of the designed cancer therapies. Although HA-based cancer therapy still has several points that should be improved by further research, the results presented in this review show the promising prospects of HA-based theranostic nanomedicine for improved targeted cancer therapies. We expect that further research using HA will provide innovative results and insights into the development of novel cancer therapies.
